# An Intelligent Nanoscale Insulin Delivery System

**DOI:** 10.3390/molecules23112945

**Published:** 2018-11-11

**Authors:** Wei Wang, Ling Liao, Xiaobing Zhang, Fan Lei, Yaou Zhang, Gan Liu, Weidong Xie

**Affiliations:** 1 Key Lab in Health Science and Technology, Division of Life Science & Health, Graduate School at Shenzhen, Tsinghua University, Shenzhen 518055, China; kappa33wei@live.com (W.W.); zxb17@mails.tsinghua.edu.cn (X.Z.); zhangyo@sz.tsinghua.edu.cn (Y.Z.); 2 Open FIESTA Center, Tsinghua University, Shenzhen 518055, China; 15602927350@163.com; 3 State Key Laboratory of Chemical Oncogenomics, Graduate School at Shenzhen, Tsinghua University, Shenzhen 518055, China; 4 School of Pharmaceutical Sciences, Tsinghua University, Beijing 100084, China; leifan@mail.tsinghua.edu.cn; 5 School of Pharmaceutical Sciences (shenzhen), Sun Yat-Sen University, Guangzhou 510275, China

**Keywords:** insulin, diabetes, drug delivery, PAM-PAspPBA-*b*-PEG

## Abstract

Insulin injection relies on strict blood glucose monitoring. However, existing techniques and algorithms for blood glucose monitoring cannot be completed in a timely way. In this study, we have developed a new intelligent glucose-sensitive insulin delivery system to stabilize blood glucose levels in the body. This system does not require real-time detection of blood glucose. First, we successfully synthesized a nanoscale material called PAM-PAspPBA-*b*-PEG by using chemical methods. We then conducted TEM, DLS, and ^1^H-NMR analyses to characterize the physicochemical properties, such as size, molecular composition, and configuration of the nanomaterial. We verified the glucose responsibility of the insulin loading nanoscale material in vitro and evaluated its safety and effect on mice in vivo. Results showed that insulin-loaded PAM-PAspPBA-*b*-PEG is glucose-sensitive, safer and more effective than regular insulin injection. This study provides a basis for future development of smart insulin delivery systems.

## 1. Introduction

The current prevalence of diabetes worldwide is high, and the number of diabetic patients is expected to increase to 592 million by 2035 [1]. Diabetes causes many serious complications and a huge economic burden to the patients’ family and society. Severe diabetes is treated by insulin injection [2]. However, current insulin injection protocols cannot fundamentally meet the actual requirements of diabetic patients because of their daily blood glucose fluctuation [3]. Insulin delivery relies on a real time-continuous glucose monitoring technology in case of hypoglycemic effects [4]. Moreover, existing glucose monitoring techniques still have many limitations.

**Figure 1:**
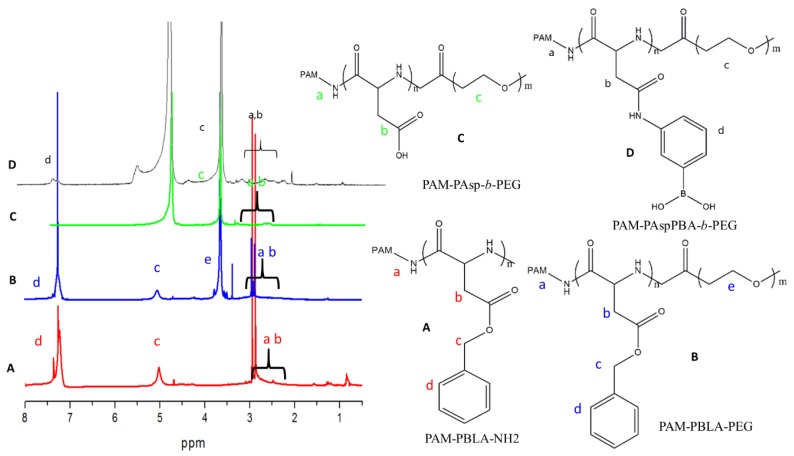
^1^H-NMR spectra of (**A**) PAM-PBLA_480_-NH_2_, (**B**) PAM-PBLA_480_-*b*-(PEG_45_)_30_, (**C**) PAM-PAsp_480_-*b*-(PEG_45_)_30_, and (**D**) PAM-(Asp_0.29_-*co*-AspPBA_0.71_)_480_-*b*-(PEG_45_)_30_.

Self-regulated insulin delivery is currently under development and has emerged as an important direction of future insulin administration [5]. This study aims to develop a new intelligent glucose-sensitive insulin delivery system that does not require real-time detection of blood glucose. We design a novel nanoscale polymer material named dendrimer polyamidoamine (PAM)-polyaspartic acid (PAsp) phenylboronic acid (PBA)-polyethylene glycol (PEG) (Figure 1). The material could package insulin in the core after self-assembly and exhibits glucose responsitivity, that is, it could mimic a physiological insulin release system when the blood glucose level changes. The self-assembled material has an excellent drug loading, as previously described [6]. Insulin is hydrophobic in the neutral solution and also can bind to the hydrophobic core of the material by Van der Waals’ force. When the material was coupled with phenylboronic acid (PBA) it demonstrated glucose responsiveness [6]. When the glucose concentration increased to some extent, the PBA contained in the material would become hydrophilic, weakening the van der Waals’ forces between the insulin and the material. The structure of the material would change and insulin might easily escape from the material, so we speculated that insulin entrapped inside the material would be easily released in a high glucose state.

## 2. Results

### 2.1. Identification of Nanoparticles

We synthesized PAM-PAspPBA-*b*-PEG in four chemical reaction steps. The chemical structure of the dendritic copolymer PAM-PAspPBA-*b*-PEG was determined by ^1^H-nuclear magnetic resonance (NMR) analysis (Figure 1). PAM-poly(β-benzyl-L-aspartate)(PBLA)-NH_2_ was synthesized according to the reference [6]. The degree of polymerization of PBLA in each PAM-PBLA-NH_2_ was calculated by comparing the peak areas of a and b with c and found to be 480 (Figure 1A). The average number of PBLA units initiated by each amine was 15 because each polyamidoamine(PAMAM)-G3 dendrimer contains 32 primary amines. As shown in Figure 1B, peak e represents the methylene of PEG (-OCH_2_CH_2_-: δ = 3.7 ppm), indicating successful grafting of PEG_45_ on the dendritic polymer. The number of PEG units grafted on each copolymer was quantified by comparing the proton ratios of the methylene units of PEG (peak e) with the methylene units of PBLA (peak c) and found to be 30, which confirms that nearly all PBLA blocks were grafted with PEG. After acid hydrolysis, the ^1^H- NMR signal that corresponds to the benzyl group and the identical degree of polymerization (DP) between PAsp and PBLA segments disappeared, indicating the complete deprotection of β-benzyl-L-aspartate (BLA) without breakage of the PAsp backbones. Finally, PAM-PAspPBA-*b*-PEG was synthesized by modifying APBA on the side -COOH group of the PAsp block. The medication degree, which is peak intensity ratio between the phenyl protons (d) and methylene protons of PEG (c), was calculated to be 71%. The molecular weight of PAM-PAspPBA-*b*-PEG was calculated as about 160 kD.

### 2.2. Characterization of Nanoparticles

**Figure 2:**
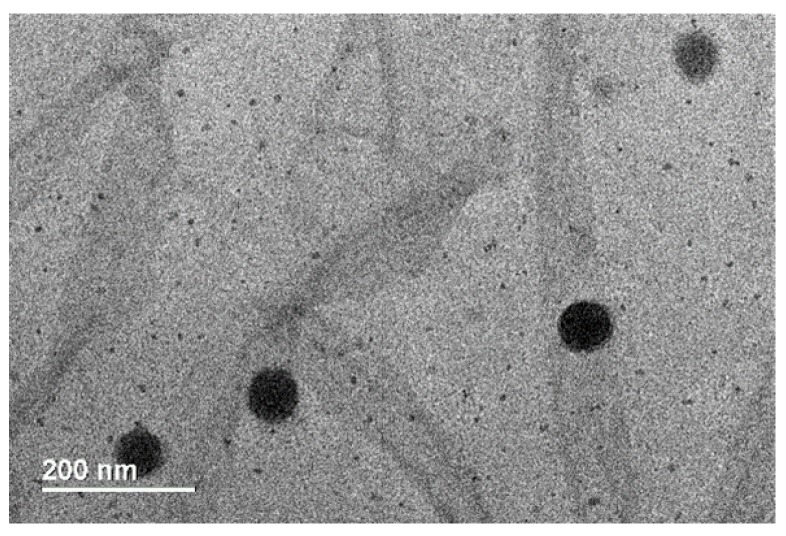
TEM image for PAM-PAspPBA-*b*-PEG.

The size of the new material PAM-PAspPBA-*b*-PEG was determined to range from 80 nm to 150 nm based on transmission electron microscopy (TE, Figure 2), which indicates that PAM-PAspPBA-*b*-PEG contains nanoparticles. The loading efficiency is 14.4% while the entrapment efficiency is 56.1%. We also tested the stability of the nanoparticle within 7 days at 4 °C, no significant difference of size was observed.

### 2.3. Glucose Response Study

**Figure 3:**
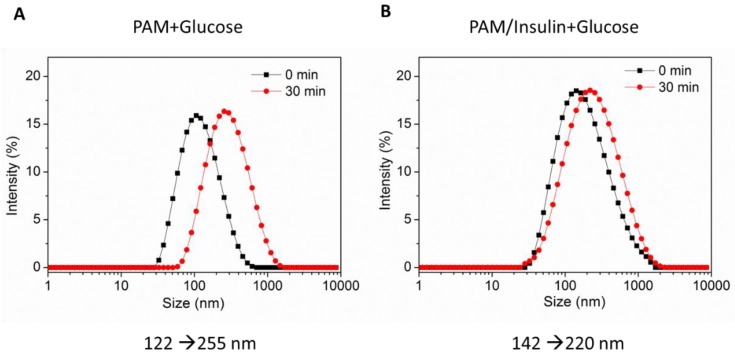
Changes of sizes of PAM-PAspPBA-b-PEG and insulin-loaded PAM-PAspPBA-*b*-PEG after adding glucose determined by DLS. PAM-PAspPBA-*b*-PEG (**A**) and insulin-loaded PAM-PAspPBA-*b*-PEG (**B**) dissolved in 20 mM glucose solution (PBS, pH = 7.4) at 0 min and 30 min.

To evaluate the glucose responsiveness of PAM-PAspPBA-*b*-PEG, dynamic light scattering (DLS) showed that the sizes of both PAM-PAspPBA-*b*-PEG and insulin-loaded PAM-PAspPBA-*b*-PEG after the addition of glucose (20 mM) for 30 min increased compared with that before the addition of glucose (Figure 3), which indicates that the nanoparticle of PAM-PAspPBA-*b*-PEG has a slight glucose responsiveness.

**Figure 4:**
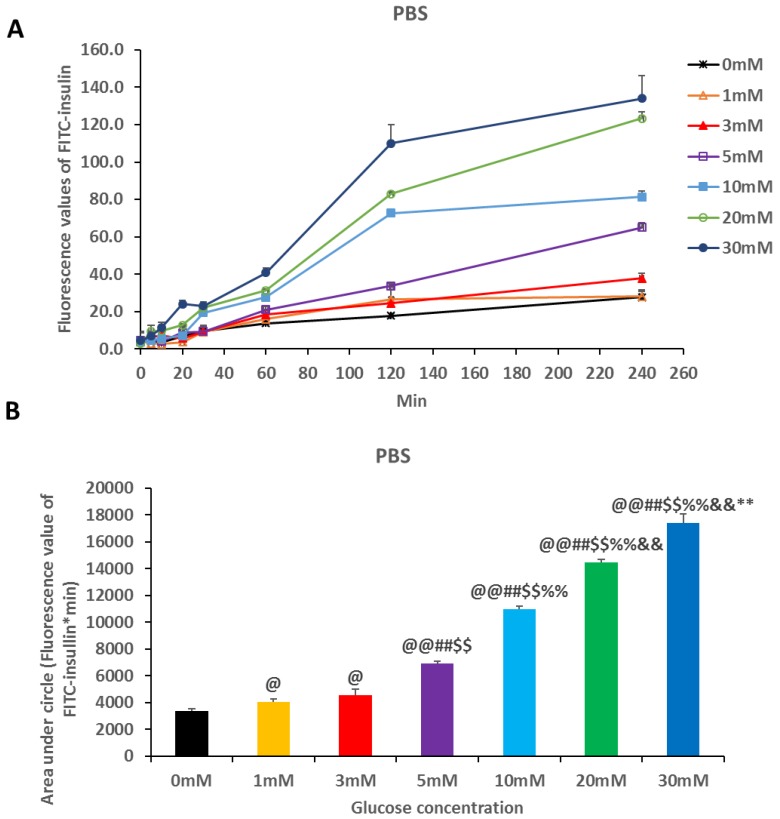
Insulin release in PBS (**A**) with different glucose concentrations. Area under circles of fluorescence values of fluorescein isothiocyanate (FITC)-insulin with different glucose concentrations within 240 min were calculated by the formulation ‘[(F0 + F5) × 5 + (F5 + F10) × 5 + (F10 + F20) × 10 + (F20 + F30) × 10 + (F30 + F60) × 30 + (F60 + F120) × 60 + (F120 + F240) × 120] × 1/2; Fn, Fluorescence value at n min, n = 0, 5, 10, 20, 30, 60, 120 or 240]’, respectively (**B**). FITC-insulin loaded PAM-PAspPBA-*b*-PEG dissolved in PBS (pH 7.4) with 0, 1, 3, 5, 10, 20 or 30 mM final glucose concentration. The experiment is repeated thrice. Data were represented as mean ± S.D.; @*P* < 0.05, @@*P* < 0.01 vs. 0 mM; ##*P* < 0.01 vs. 1 mM; $$*P* < 0.01 vs. 3 mM; %%*P* < 0.01 vs. 5 mM; &&*P* < 0.01 vs. 10 mM; **P < 0.01 vs. 20 mM.

**Figure 5:**
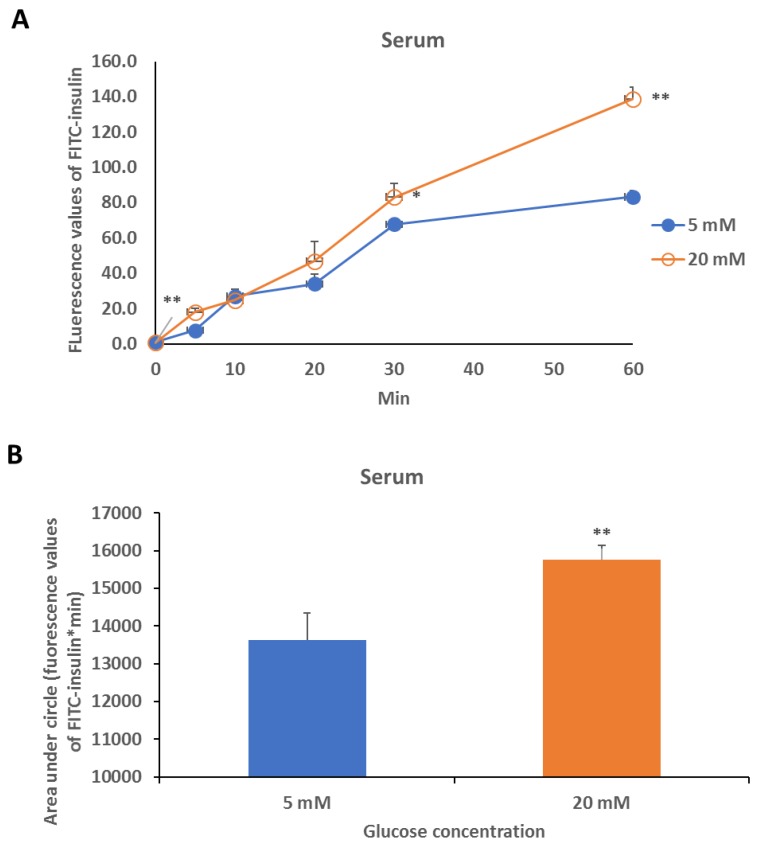
Insulin release in serum (**A**) with different glucose concentrations. Area under circles of fluorescence values of FITC-insulin within 60 min in serum were calculated by the formulation ‘[(F0 + F5) × 5 + (F5 + F10) × 5 + (F10 + F20) × 10 + (F20 + F30) × 10+(F30 + F60) × 30] × 1/2; Fn, Fluorescence value at n min, n = 0, 5, 10, 20, 30 or 60]’ (**B**). FITC-insulin-loaded PAM-PAspPBA-*b*-PEG dissolved in serum (pH 7.4) with 5 mM or 20 mM final glucose concentration. The experiment is repeated thrice. Data were represented as mean ± S.D., *P < 0.05, **P < 0.01 vs. 5 mM (or at respective time points, see **A**).

Furthermore, we used phosphate buffer saline (PBS) and fetal bovine serum (FBS) that contained different glucose concentrations (0–30 mM), and we determined that the insulin release in the solutions. We found that glucose dramatically increased insulin release in PBS solution contained glucose almost in a dose-dependent manner (Figure 4A,B). Also, high concentration of glucose showed a significant increase in insulin release in the serum compared with the low concentration of glucose (Figure 5A,B).

### 2.4. Safety and Effect Evaluation

**Figure 6:**
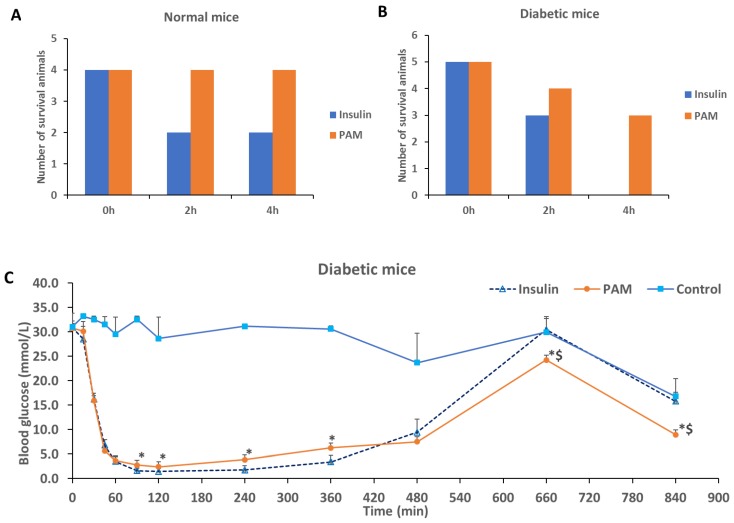
Safety (**A** and **B**) and effect (**C**) evaluation of insulin-loaded PAM-PAspPBA-*b*-PEG in normal and diabetic mice. Pure insulin-treated (Insulin) and insulin-loaded PAM-PAspPBA-*b*-PEG-treated (PAM) animals received a subcutaneous injection of insulin at identical dosage of 0.25 (for effect experiment) or 1.2 mg/kg (for safety experiment), *n* = 4–5. For diabetic mice, data were represented as mean ± S.D, *P < 0.05, **P < 0.01 vs. Insulin, ^$^*P* < 0.05 vs. diabetic control (Control).

To test the safety of the insulin-loaded PAM-PAspPBA-*b*-PEG in mice, we determined that the injection of insulin-loaded PAM-PAspPBA-*b*-PEG did not cause death in the normal mice, whereas the injection of pure insulin at identical dosage caused the death of 50% mice due to hypoglycemia (Figure 6A). In the diabetic mice, there were no survivals for pure insulin treatment while insulin-loaded PAM-PAspPBA-*b*-PEG at identical dosage could cause 60% mice survived.

Subsequently, we evaluated whether the insulin-loaded PAM-PAspPBA-*b*-PEG had a similar effect to pure insulin in diabetic mice in hyperglycemic state. There was no significant difference observed between two groups within 60 min of the injection, an early stage after insulin injection (Figure 6C). However, pure insulin injection had a lower glucose level compared with insulin-loaded PAM-PAspPBA-*b*-PEG at 90 and 120 min of the injection, which might explain why pure insulin treated mice were easily dead caused by severe hypoglycemic response. Intriguingly, in the late stage of injection, insulin-loaded PAM-PAspPBA-*b*-PEG still showed significant decrease in blood glucose while pure insulin treatment did not exert glucose-lowering effects at 660 and 840 min. Obviously, the insulin-loaded PAM-PAspPBA-*b*-PEG at identical insulin dosage had a better blood glucose control ability compared with pure insulin in diabetic mice.

### 2.5. Biocompatibility Tests

**Figure 7:**
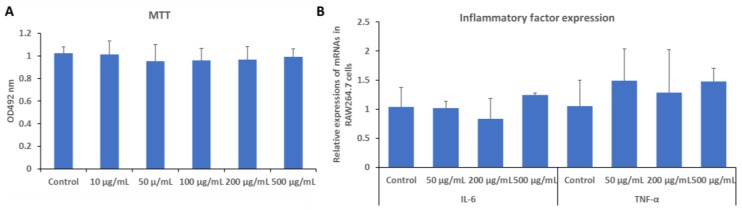
Cytotoxicity assay (**A**) by MTT method and inflammatory factor expressions (**B**) by RT-PCR analysis in RAW264.7 cells.

We conducted biocompatibility tests by cytotoxicity experiments in RAW264.7 cells. There was no significant cytotoxicity observed after adding PAM-PAspPBA-*b*-PEG within 500 µg/mL (final concentration, Figure 7A) by using a 3-(4,5-dimethylthiazol-2-Yl)-2,5-diphenyltetrazolium bromide (MTT) assay. 

Also, we did not see significant changes of inflammatory factor expression after exposing into PAM-PAspPBA-*b*-PEG (Figure 7B) by RT-PCR analysis. Considering that the dosage of insulin was not more than 24 IU (about 1 mg) per day [6], according to the loading efficiency of insulin (14.4%), in human, the estimated dosage of PAM-PAspPBA-*b*-PEG was not more than 6 mg per day. The blood volume of an adult was usually 4–5 L, so the estimated concentrations of PAM-PAspPBA-*b*-PEG were not more than 2 µg/mL per day in blood, which may be far less than 500 µg/mL. Furthermore, the micelle contained poly(amino acids) that would be degraded and metabolized gradually. Taken together, there is no indication that the micelles cause toxicity or inflammatory stimulation responses and they seem safe to serve as a vehicle.

## 3. Discussion

Type 1 diabetes and severe type 2 diabetes depend on insulin injection. However, insulin injection on those patients is difficult because the rapid detection of blood glucose is complicated. Self-regulated insulin delivery systems that simulate the physiological response are well-developed [7]. Synthetic PBA-based polymeric particles have better stability and less immunogenicity than glucose oxidase and lectin and have received considerable attention [8].

Previous studies have proved that the self-assembled PBA-based polymeric micelles from poly- (ethylene glycol)-block-poly(acrylic acid-co-acrylamidophenylboronic acid) (PEG-*b*-(PAA-*co*-PAAPBA)) exhibit glucose-responsive release of insulin under physiological conditions [9]. However, a simple core–shell structure cannot achieve repeated on–off release due to its disassembly in response to glucose. Then, self-assembled complex micelles from two types of diblock copolymers, namely, poly(ethylene glycol)-*b*-poly(aspartic acid-co-aspartamidophenylboronic acid and poly(*N*-isopropylacrylamide)-*b*-poly(aspartic acid-co-aspartamidophenylboronic acid) [6], have been developed to achieve repeated glucose-responsive on–off insulin release. However, they still require optimization due to their complicated synthesis and low biocompatibility.

Dendritic polymeric micelles fabricated from multiarm star block copolymers have attracted considerable attentions due to their excellent in vivo stability and other advantages, such as no surrendered degradability and drug release profiles and narrow disperse dimensions. PAMAM dendrimers, which are the first commercialized dendrimer family, have good biocompatibility, structural control, functionalizability, and low cost, which are usually used in nanotechnology and drug development [10,11,12]. They are widely utilized as macroinitiators in synthesizing dendritic polymeric micelles due to their numerous tailorable terminal functional groups. In this study, we synthesized a new polymeric material named PAM-PAspPBA-*b*-PEG based on PAMAMs through a simple chemical synthesis. The core–shell structure has considerable insulin load, good biocompatibility, and low cost. Obviously, the insulin-loaded PAM-PAspPBA-*b*-PEG showed comparable glucose responsiveness.

Regular insulin injection can easily cause a hypoglycemic event, shock, and even death if blood glucose cannot be closely monitored. Approximately 25% diabetic American patients have a low blood glucose level upon hospitalization [13]. Here, we determined that insulin-loaded PAM-PAspPBA-*b*-PEG did not easily cause a hypoglycemic death in mice due to low insulin release, which indirectly indicates that the insulin-loaded PAM-PAspPBA-*b*-PEG is safer. This result in vivo enhances a concept that insulin-loaded PAM-PAspPBA-*b*-PEG has less insulin release compared with pure insulin when blood glucose is low. However, we determined that the insulin-loaded PAM-PAspPBA*-b*-PEG has a similar effect to pure insulin in a hyperglycemic state, which indirectly indicates that insulin-loaded PAM-PAspPBA-*b*-PEG has similar release to pure insulin in the case of hyperglycemia. Blood glucose fluctuation can cause serve damage to the human body in diabetic individuals [14]. Intriguingly, insulin-loaded PAM-PAspPBA-*b*-PEG has a better effect on preventing the development of hypoglycemia or hyperglycemia than pure insulin in diabetic animals, which suggests that insulin-loaded PAM-PAspPBA-*b*-PEG might have a better control over blood glucose fluctuation than pure insulin and show a promising application in diabetic individuals.

## 4. Materials and Methods

### 4.1. Materials

Dendrimer PAMAM G3 was purchased from Weihai CY Dendrimer Technology Co. Ltd., China. β-Benzyl-L-aspartate N-carboxyanhydride (BLA-NCA) was obtained from Nanjing Chemlin Chemical Industry Co., Ltd. (Nanjing, China). PEG-SCM was provided by Wuhu Ponsure Biotechnology Co. Ltd. (Wuhu, China). 3-Aminophenylboronic acid (APBA), trifluoroacetic acid (CF_3_COOH), *N*,*N*-dimethylformamide (DMF), and *N*-(3-dimethylaminopropyl)-*N*′-ethyl-carbodiimide hydrochloride (EDC) were purchased from Sigma-Aldrich (St. Louis, MO, USA). 3-(4,5-Dimethylthiazol-2-yl)-2,5-diphenyltetrazolium bromide (MTT) was purchased from Amresco (Radnor, OH, USA). Insulin was obtained from Beijing Dingguo Changsheng Biotechnology Co. Ltd. (Beijing, China). FBS was purchased from PAN-Biotech (Aidenbach, German). Dulbecco′s Modified Eagle Medium (DMEM) was purchased from Thermo Fisher Scientific (Waltham, MA, USA).

### 4.2. Synthesis of Polymer

#### 4.2.1. Synthesis of PAM-PBLA-NH_2_

First, PAM-PBLA-NH_2_ was synthesized by anionic polymerization reaction between PAMAM-G3-NH_2_ and BLA-NCA. Briefly, BLA-NCA (3 g) was dissolved in the mixture solution of DMF (30 mL) and dichloromethane (CH_2_Cl_2_, 3 mL) at 80 °C. Then, PAMAM-G3-NH_2_ solution (0.9 g, dissolved in 20% methanol) was added in BLA-NCA solution. The reaction mixture was stirred for 3 days at 25 °C under dry nitrogen atmosphere. Subsequently, the reaction mixture was precipitated in 10-fold excess of precooled (−20 °C) diethyl ether (300 mL). Then, the diethyl ether in the solution was evaporated to driness, and the remaining precipitate was PAM-PBLA-NH_2_.

#### 4.2.2. Synthesis of PAM-PBLA-PEG

Second, PAM-PBLA-PEG was synthesized by amidation reaction between PAM-PBLA-NH_2_ and PEG-SCM. Briefly, PAM-PBLA-NH_2_ (1 g) was dissolved in DMF (10 mL). Then, PEG-SCM (1 g) was added in PAM-PBLA-NH_2_ solution. The reaction mixture was stirred for 2 h at room temperature. Subsequently, the reaction mixture solution was precipitated in 10-fold excess of precooled (4 °C) diethyl ether (100 mL). After overnight precipitation at 4 °C, the precipitate was obtained by centrifugation at 3000 rpm for 5 min. The crude precipitate was washed twice with diethyl ether to obtain the precipitate. The precipitate was dried by vacuum evaporation to obtain PAM-PBLA-PEG.

#### 4.2.3. Synthesis of PAM-PAsp-*b*-PEG

Third, PAM-PAsp-*b*-PEG was obtained by hydrolysis reaction between PAM-PBLA-PEG and CF_3_COOH. Briefly, PAM-PBLA-PEG (1 g) was dissolved in CF_3_COOH (10 mL). Hydrogen bromide (HBr)/acetic acid (HAc) (1:2, *v*/*v*; 1 mL) was added to PAM-PBLA-PEG solution. The reaction mixture was stirred for 1 h at room temperature. Subsequently, the reaction mixture was precipitated in 10-fold excess of precooled (4 °C) diethyl ether (200 mL). After overnight precipitation at 4 °C, the precipitate was obtained by centrifugation at 3000 rpm for 5 min. The precipitate was dissolved in deionized water and was dialyzed against water for 2 days in a dialysis bag with a molecular cutoff of 5 kD. The solution was lyophilized to obtain PAM-PAsp-b-PEG.

#### 4.2.4. Synthesis of PAM-PAspPBA-*b*-PEG

**Scheme 1:**
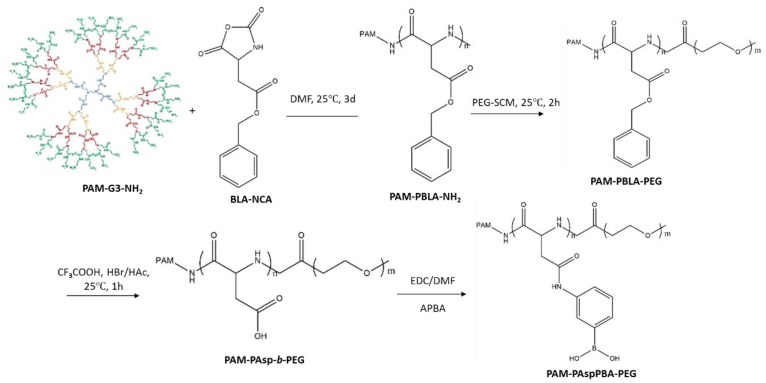
Synthesis scheme of PAM-PAspPBA-*b*-PEG.

Fourth, PAM-PAspPBA-*b*-PEG was synthesized by amidation reaction between PAM-PAsp-b-PEG and APBA. Briefly, PAM-PAsp-*b*-PEG (0.5 g) and APBA (0.305 g) were dissolved in DMF (10 mL). EDC (2 g) was added to the solution. The mixture solution was magnetically stirred for 4 h at 4 °C. Subsequently, a 10-fold volume of deionized water (pH = 3) was added, and the precipitate was isolated by centrifugation at 3000 rpm for 10 min. The precipitate was washed with deionized water (pH 7.0) and was lyophilized to obtain PAM-PAspPBA-*b*-PEG. The synthesis scheme of PAM-PAspPBA-*b*-PEG is summarized in Scheme 1.

### 4.3. Preparation of FITC-Insulin Loaded Micelles

Fifth, insulin (20 mg) was dissolved in deionized water (1 mL, pH = 2). FITC (2 mg) was dissolved in dimethyl sulfoxide (DMSO, 0.2 mL). Insulin and FITC solutions were mixed for 12 h at 4 °C. FITC-insulin was produced and was dialyzed against deionized water for 2 days and was lyophilized. PAM-PAspPBA-*b*-PEG (20 mg) was dissolved in PBS (pH = 12) and FITC-insulin (2 mg) was dissolved in PBS (pH = 2). FITC-insulin solution was added to PAM-PAspPBA-*b*-PEG solution at a rate of six drops per minute until the pH becomes 6. A Tyndall effect was observed, and FITC-insulin loading PAM-PAspPBA-*b*-PEG micelles were obtained. These insulin-loading micelles were dialyzed against deionized water for 2 days in a dialysis bag with a molecular cutoff of 5 kD.

### 4.4. Characterization of Micelles

#### 4.4.1. ^1^H-NMR

^1^H-NMR spectra were obtained on a UNITY-plus 400 M nuclear magnetic resonance spectrometer (Varian, Palo Alto, CA, USA) using chloroform-d (CDCl_3_), heavy water (D_2_O), and deuterated sodium hydroxide (NaOD) as solvents. Gel permeation chromatography (GPC) measurements of block copolymers were conducted at 35 °C by using a Waters 1515 chromatography system (Waters, Milford, MA, USA) equipped with a Waters 2414 refractive index detector. Tetrahydrofuran (THF) and DMF containing 500 mM lithium bromide (LiBr) were separately used as eluent at a flow rate of 1 mL min^−1^. Polystyrene standards were employed in calibration.

#### 4.4.2. TEM Measurements

TEM measurements were performed by using a commercial T20ST electron microscope (Philips, Amsterdam, Holland) at an acceleration voltage of 200 kV. To prepare the TEM samples, a small drop of the micellar solution was deposited on a carbon-coated copper electron microcopy grid and was dried under room temperature and atmospheric pressure.

#### 4.4.3. DLS measurements

The structure of the micelles was characterized by DLS measurements. In this study, DLS measurements were performed at 636 nm by using a laser light scattering spectrometer (BI-200SM, Brookhaven, New York, NY, USA) equipped with a digital correlator (BI-9000AT).

#### 4.4.4. Insulin Release Study

About 0.2–1 mL of FITC-Insulin-loaded micelle solution (1 mg/mL, calculated by PAM-PAspPBA-*b*-PEG) was injected in a dialysis bag with a molecular weight cutoff of 10 kD, and the dialysis bag was placed in 10–30 mL of PBS (pH 7.4) with different glucose concentrations (0, 1, 3, 5, 10, 20 and 30 mM). About 200 µL of the dialysis solution was used to measure the insulin concentration at different time points (0, 5, 10, 20, 30, 60, 120 and 240 min) by assaying fluorescence intensity (excitation wavelength: 490 nm, emission wavelength: 525 nm). This experiment was repeated thrice. Once we took out the dialysis solution for assay, identical volume of PBS should be immediately added to the dialysis solution. We also evaluated the insulin release of the micelle in serum (100% FBS, pH 7.4) with different glucose concentrations (5 and 20 mM) at different time points (0, 5, 10, 20, 30 and 60 min) as described above.

#### 4.4.5. Safety and Effect Evaluation in Animals

Male NIH mice (4-week old) were purchased from Guangdong Medical Animal Center (Guangzhou, China). The animals were kept at a room with temperature 20 ± 2 °C, humidity 60% ± 5%, and 12 h dark/light cycle. All experiments were strictly in accordance with the recommendations of the Guide for the Care and Use of Laboratory Animals of the Institutional Animal Care and Use Committee of Tsinghua University (No.17-lf1#). For the safety assay, after acclimation for two weeks, the normal animals were randomly divided into two groups (four mice per group). The first group was subcutaneously injected with insulin-loaded PAM-PAspPBA-*b*-PEG that contained 1.2 mg/kg insulin. The second group was subcutaneously injected with pure insulin at a dosage of 1.2 mg/kg. The normal animals were analyzed in a fasting condition. The number of animal survivals was recorded after 2 and 4 h of injection. For diabetic mice, after acclimation for one week, diabetes was induced in mice fasting for 24 h by intraperitoneal injection of streptozotocin (Sigma-Aldrich, St. Louis, MO, USA; 100 mg/kg) according to the previous method [15]. After a week, mice with blood glucose over 11.1 mmol/L were selected as the diabetic model and subjected into further investigation. The protocol of the safety assay in diabetic mice (five mice per group) was similar to that in normal mice.

For the effect assay, the selected diabetic mice were divided into three groups, where the first group was subcutaneously injected with insulin-loaded PAM-PAspPBA-*b*-PEG that contained 0.25 mg/kg insulin, the second group was treated with pure insulin at a dosage of 0.25 mg/kg and the third group was treated with PBS control. In the preliminary experiment, pure PAM-PAspPBA-*b*-PEG did not affect blood glucose (data not shown). The diabetic animals were analyzed in a fasting state. Blood glucose was assayed at 0, 15, 30, 45, 60, 90, 120, 240, 360, 480, 660 and 840 min by using a glucometer (Roche, Basel, Switzerland).

### 4.5. Cytotoxicity and Inflammatory Factor Assays

The cytotoxicity assay of PAM-PAspPBA-*b*-PEG was conducted in RAW264.7 cells by MTT method as described previously [6]. Briefly, RAW264.7 cells were seeded into 96-well plate at cell density of 10,000 per well and incubated with DMEM plus 10% FBS. PAM-PAspPBA-*b*-PEG resuspended in DMEM at final concentrations of 10, 50, 100, 200 and 500 µg/mL, respectively, were added into the cells. After 24 h of incubation, 20 µL of MTT solution (5 mg/mL in PBS buffer) was added to each well. Four hours later, the medium was removed and the samples in the wells were washed with PBS twice. About 100 µL of DMSO was added to dissolve the formed crystals for 30 min. The optical density of the solution was measured at 492 nm using a microplate reader (BioTek Instruments, Inc., Winooski, VT, USA).

In addition, inflammatory factor (IL-6 and TNF-α) expressions were determined by RT-PCR method as described previously [16]. Briefly, RAW264.7 cells were seeded into 6-well plate at cell density of 250,000 per well and incubated with DMEM plus 10% FBS. PAM-PAspPBA-*b*-PEG resuspended in DMEM at final concentrations of 50, 200 and 500 µg/mL, respectively, were added into the cells. After 24 h of incubation, total RNA was extracted by using TRIzol reagent (Thermo Fisher Scientific, Waltham, MA, USA) and conduct RT-PCR analysis by using a commercial kit (Takara, Beijing, China). Primers of IL-6 (Forward: CTGCAAGAGACTTCCATCCAG; Reverse GAGTGGTATAGACAGGTCTGTTGG), TNF-α (Forward: GGGCTTCCAGAACTCCA; Reverse: GCTACAGGCTTGTCACTCG), and actin (Forward: GTGACGTTGACATCCGTAAAGA; Reverse: GCCGGACTCATCGTACTCC) were purchased from Thermo Fisher Scientific (Waltham, MA, USA). Actin was used to normalize the values of IL-6 and TNF-α. Relative expressions were calculated as 2^−ddct^.

### 4.6. Statistical Analysis

Data were expressed as mean ± SD. Statistical significance was evaluated through one-way ANOVA and set as *P* < 0.05. When appropriate, Newman-Keuls comparison was used to determine the source of significant differences.

## 5. Conclusions

We successfully synthesized a new material called PAM-PAspPBA-*b*-PEG. The material showed a nanoparticle characterization. The insulin-loaded PAM-PAspPBA-*b*-PEG nanoparticle is glucose responsive, more effective, and safer than pure insulin. This study provides guidance for the future development of smart insulin delivery systems.
